# [1,3-Bis(diphenyl­phosphino)pentane-κ^2^ *P*,*P*′]tetra­carbonyl­chromium(0)

**DOI:** 10.1107/S1600536809001202

**Published:** 2009-02-04

**Authors:** Omar bin Shawkataly, Daniel T. Thangadurai, Mohd. Aslam A. Pankhi, S. M. Shahinoor Dulal Islam, Hoong-Kun Fun

**Affiliations:** a Chemical Sciences Programme, Centre for Distance Education, Universiti Sains Malaysia, 11800 USM, Penang, Malaysia; b X-Ray Crystallography Unit, School of Physics, Universiti Sains Malaysia, 11800 USM, Pulau Pinang, Malaysia

## Abstract

In the title compound, [Cr(C_29_H_30_P_2_)(CO)_4_], the Cr atom is octa­hedrally coordinated by four carbonyl ligands and one bidentate phosphine ligand, which is bounded as a chelate in a *cis* position. The average Cr—P and Cr—C bond lengths are 2.377 and 1.865 Å, respectively.

## Related literature

For chromium–carbonyl complexes see: Shawkataly *et al.* (1996 ▶, 1997 ▶, 2006 ▶); for Cr—C bond lengths see: Bennett *et al.* (1971 ▶); Ueng & Shih (1992 ▶). For Cr—C and C—O distances see Whitaker & Jeffery (1967 ▶); Jost *et al.* (1975 ▶). For a description of the Cambridge Structural Database, see: Allen (2002 ▶).
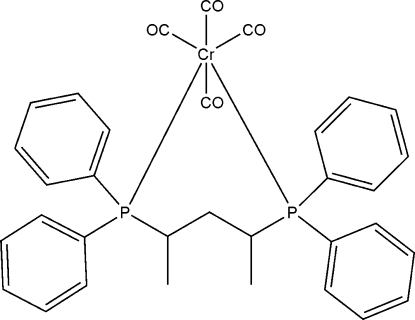


## Experimental

### 

#### Crystal data

[Cr(C_29_H_30_P_2_)(CO)_4_]
*M* *_r_* = 604.51
Orthorhombic, 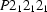

*a* = 13.3013 (2) Å
*b* = 14.2333 (2) Å
*c* = 15.6694 (3) Å
*V* = 2966.55 (8) Å^3^
*Z* = 4
Mo *K*α radiation
μ = 0.53 mm^−1^
*T* = 293 (2) K
0.48 × 0.42 × 0.28 mm

#### Data collection

Siemens SMART CCD diffractometer
Absorption correction: multi-scan (*SADABS*; Sheldrick, 2001 ▶) *T*_min_ = 0.785, *T*_max_ = 0.866
24593 measured reflections
7364 independent reflections
6364 reflections with *I* > 2σ(*I*)
*R*_int_ = 0.049

#### Refinement

*R*[*F* ^2^ > 2σ(*F* ^2^)] = 0.031
*wR*(*F* ^2^) = 0.072
*S* = 1.03
7364 reflections
361 parameters
H-atom parameters constrained
Δρ_max_ = 0.19 e Å^−3^
Δρ_min_ = −0.30 e Å^−3^
Absolute structure: Flack (1983 ▶), 3256 Friedel pairs
Flack parameter: −0.001 (13)

### 

Data collection: *SMART* (Siemens, 1994 ▶); cell refinement: *SAINT* (Siemens, 1994 ▶); data reduction: *SAINT*; program(s) used to solve structure: *SHELXS86* (Sheldrick, 2008 ▶); program(s) used to refine structure: *SHELXL97* (Sheldrick, 2008 ▶); molecular graphics: *ORTEP-3 for Windows* (Farrugia, 1997 ▶); software used to prepare material for publication: *WinGX* (Farrugia, 1999 ▶).

## Supplementary Material

Crystal structure: contains datablocks I, global. DOI: 10.1107/S1600536809001202/kj2106sup1.cif

Structure factors: contains datablocks I. DOI: 10.1107/S1600536809001202/kj2106Isup2.hkl

Additional supplementary materials:  crystallographic information; 3D view; checkCIF report

## Figures and Tables

**Table:** 

Cr1—C1	1.851 (2)
Cr1—C2	1.8650 (19)
Cr1—C3	1.872 (2)
Cr1—C4	1.901 (2)
Cr1—P2	2.3736 (5)
Cr1—P3	2.3847 (5)

**Table:** 

P2—Cr1—P3	91.389 (18)

**Fig. 1.**
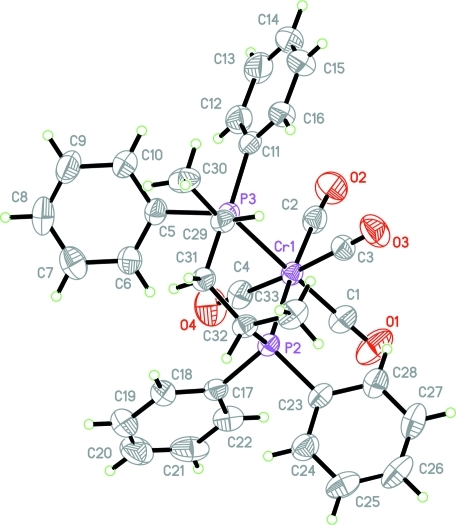
View of the title compound (50% probability displacement ellipsoids).

## supplementary crystallographic information

### Comment

It is generally believed that the metal (*M*) to carbon monoxide bond involves both OC—*M*σ-bonding and M—CO π-bonding. In view of this phenomenon, the bonding characteristics of metal carbonyls with a phosphine ligand in phosphine-substituted metal carbonyls are of interest. A search of the Cambridge Structural Database (Version 5.29; Allen, 2002) revealed only 88 complexes of group VI metal carbonyls with a 3-carbon backbone bidentate phosphine. However, there are only a few examples of chromium carbonyl complexes (Shawkataly *et al.*, 2006). Previously, we reported several crystal structures of phosphine-substituted group VI metal carbonyls (Shawkataly *et al.*, 1996,1997). We present here the crystal structure of the title compound.

The title compound has an expected octahedral geometry (Fig. 1). The Cr—C bond lengths of the *cis* carbonyl ligands (with respect to the P atom) are slightly longer than those for the *trans* carbonyl group (Table 1). This trend was also observed in Cr[Ph_2_P(CH_2_)_2_PPh_2_](CO)_4_ (Bennett *et al.*, 1971) and Cr[Ph_2_P(CH_2_)_4_PPh_2_](CO)_4_ (Ueng & Shih, 1992). The bidentate phosphine bite angle [91.389 (18)°] is intermediate between that observed in Cr[Ph_2_P(CH_2_)_2_PPh_2_](CO)_4_ (83.41 (8)°] and that in Cr[Ph_2_P(CH_2_)_4_PPh_2_](CO)_4_ (93.29 (5)°]. Comparison of the mean Cr—C and C—O distances in the title compound [1.872 (2) and 1.145 (6) Å, respectively] with those in Cr(CO)_6_ [1.909 (3) and 1.137 (4) Å, respectively (Whitaker & Jeffery, 1967); and 1.918 (2) and 1.141 (2) Å, respectively (Jost *et al.*, 1975)], indicates stronger bonding owing to the back-bondi ng abilities of the bidentate phosphine. The Cr—P bond lengths, with an average values of 2.3792 (5) Å, are relatively short inspite of the presence of the bulky phosphine ligand.

### Experimental

A mixture of Cr(CO)_6_ (1.064 mmol) and Ph_2_P(CH_3_)CH(CH_2_)CH(CH_3_)PPh_2_ (1.065 mmol) was refluxed in a purified mixture of petroleum ether (60–80 °C, 25 ml) and butanol (20 ml) for *ca* 12 h under nitrogen atmosphere. The solvent was evaporated and the crude product was dissolved in acetone (5 ml) and filtered. Yellow crystals (75% yield) were obtained by slow evaporation of the acetone solution at room temperature. Analysis calculated for C_33_H_30_CrO_4_P_2_: C 65.55, H 5.01%; found C 65.54, H 5.00%.

### Refinement

All H atoms were placed at calculated positions and refined using a riding model, with C—H = 0.93–0.98Å, C—H= 0.97 Å (methylene) and C—H= 0.96 Å (methyl) and *U*_iso_(H) = 1.2*U*_eq_(C, aromatic, methylene) and *U*_iso_(H)=1.5U_equ_(C methyl). A rotating group model was used for the methyl group. The number of Friedel pairs are 3260.

### Crystal data

**Table:**

[Cr(C_29_H_30_P_2_)(CO)_4_]	*F*(000) = 1256
*M**_r_* = 604.51	*D*_x_ = 1.354 Mg m^−^^3^
Orthorhombic, *P*2_1_2_1_2_1_	Mo *K*α radiation, λ = 0.71073 Å
Hall symbol: P 2ac 2ab	Cell parameters from 427 reflections
*a* = 13.3013 (2) Å	θ = 2–27.5°
*b* = 14.2333 (2) Å	µ = 0.53 mm^−^^1^
*c* = 15.6694 (3) Å	*T* = 293 K
*V* = 2966.55 (8) Å^3^	Prism, yellow
*Z* = 4	0.48 × 0.42 × 0.28 mm

### Data collection

**Table:**

Siemens SMART CCD diffractometer	7364 independent reflections
Radiation source: fine-focus sealed tube	6364 reflections with *I* > 2σ(*I*)
graphite	*R*_int_ = 0.049
ω scans	θ_max_ = 28.3°, θ_min_ = 1.9°
Absorption correction: multi-scan (*SADABS*; Sheldrick, 2001)	*h* = −17→12
*T*_min_ = 0.785, *T*_max_ = 0.866	*k* = −18→18
24593 measured reflections	*l* = −18→20

### Refinement

**Table:**

Refinement on *F*^2^	Secondary atom site location: difference Fourier map
Least-squares matrix: full	Hydrogen site location: inferred from neighbouring sites
*R*[*F*^2^ > 2σ(*F*^2^)] = 0.031	H-atom parameters constrained
*wR*(*F*^2^) = 0.072	*w* = 1/[σ^2^(*F*_o_^2^) + (0.0349*P*)^2^] where *P* = (*F*_o_^2^ + 2*F*_c_^2^)/3
*S* = 1.03	(Δ/σ)_max_ = 0.001
7364 reflections	Δρ_max_ = 0.19 e Å^−^^3^
361 parameters	Δρ_min_ = −0.29 e Å^−^^3^
0 restraints	Absolute structure: Flack (1983), 3256 Friedel pairs
Primary atom site location: structure-invariant direct methods	Flack parameter: −0.001 (13)

### Special details

**Table:**

Geometry. All e.s.d.'s (except the e.s.d. in the dihedral angle between two l.s. planes) are estimated using the full covariance matrix. The cell e.s.d.'s are taken into account individually in the estimation of e.s.d.'s in distances, angles and torsion angles; correlations between e.s.d.'s in cell parameters are only used when they are defined by crystal symmetry. An approximate (isotropic) treatment of cell e.s.d.'s is used for estimating e.s.d.'s involving l.s. planes.
Refinement. Refinement of *F*^2^ against ALL reflections. The weighted *R*-factor *wR* and goodness of fit *S* are based on *F*^2^, conventional *R*-factors *R* are based on *F*, with *F* set to zero for negative *F*^2^. The threshold expression of *F*^2^ > σ(*F*^2^) is used only for calculating *R*-factors(gt) *etc*. and is not relevant to the choice of reflections for refinement. *R*-factors based on *F*^2^ are statistically about twice as large as those based on *F*, and *R*- factors based on ALL data will be even larger.

### Fractional atomic coordinates and isotropic or equivalent isotropic displacement parameters (Å^2^)

**Table:**

	*x*	*y*	*z*	*U*_iso_*/*U*_eq_	
Cr1	0.21688 (2)	0.11992 (2)	0.931300 (16)	0.03170 (7)	
P2	0.33880 (3)	0.10714 (3)	0.82130 (3)	0.03095 (10)	
P3	0.10424 (3)	0.01926 (3)	0.85607 (3)	0.03203 (10)	
O1	0.35830 (13)	0.24334 (14)	1.02996 (12)	0.0745 (5)	
O2	0.07737 (12)	0.14004 (13)	1.08126 (9)	0.0648 (5)	
O3	0.29780 (14)	−0.05494 (12)	1.01576 (10)	0.0644 (4)	
O4	0.11867 (14)	0.29523 (11)	0.85872 (12)	0.0696 (5)	
C1	0.30535 (15)	0.19547 (15)	0.99132 (13)	0.0448 (5)	
C2	0.12769 (14)	0.13194 (15)	1.02249 (12)	0.0434 (5)	
C3	0.26815 (15)	0.01112 (15)	0.98236 (11)	0.0417 (4)	
C4	0.15612 (16)	0.22871 (15)	0.88349 (12)	0.0436 (5)	
C5	0.01810 (14)	0.06112 (14)	0.77239 (11)	0.0382 (4)	
C6	0.05328 (15)	0.12712 (14)	0.71520 (12)	0.0425 (4)	
H6	0.1137	0.1577	0.7262	0.051*	
C7	0.00026 (19)	0.14868 (16)	0.64175 (14)	0.0573 (6)	
H7	0.0263	0.1915	0.6028	0.069*	
C8	−0.09146 (19)	0.10630 (19)	0.62657 (14)	0.0642 (7)	
H8	−0.1276	0.1210	0.5776	0.077*	
C9	−0.12896 (18)	0.0428 (2)	0.68371 (15)	0.0625 (7)	
H9	−0.1911	0.0150	0.6736	0.075*	
C10	−0.07513 (16)	0.01932 (17)	0.75678 (14)	0.0513 (5)	
H10	−0.1012	−0.0241	0.7952	0.062*	
C11	0.01881 (13)	−0.03718 (14)	0.93242 (12)	0.0390 (4)	
C12	−0.06244 (15)	0.01504 (18)	0.96266 (13)	0.0523 (5)	
H12	−0.0752	0.0743	0.9401	0.063*	
C13	−0.12452 (17)	−0.0206 (2)	1.02619 (15)	0.0691 (8)	
H13	−0.1788	0.0144	1.0459	0.083*	
C14	−0.10527 (18)	−0.1081 (2)	1.05985 (14)	0.0720 (8)	
H14	−0.1470	−0.1322	1.1022	0.086*	
C15	−0.0251 (2)	−0.1601 (2)	1.03155 (14)	0.0639 (7)	
H15	−0.0126	−0.2190	1.0548	0.077*	
C16	0.03757 (16)	−0.12478 (16)	0.96805 (12)	0.0475 (5)	
H16	0.0922	−0.1600	0.9494	0.057*	
C17	0.32879 (14)	0.20310 (13)	0.74299 (11)	0.0356 (4)	
C18	0.29810 (16)	0.19096 (15)	0.65920 (13)	0.0486 (5)	
H18	0.2886	0.1307	0.6378	0.058*	
C19	0.2816 (2)	0.26759 (18)	0.60726 (16)	0.0643 (6)	
H19	0.2594	0.2584	0.5516	0.077*	
C20	0.29715 (19)	0.35629 (19)	0.63617 (18)	0.0676 (7)	
H20	0.2856	0.4074	0.6006	0.081*	
C21	0.33051 (18)	0.37031 (16)	0.71937 (18)	0.0620 (6)	
H21	0.3426	0.4308	0.7392	0.074*	
C22	0.34562 (16)	0.29421 (14)	0.77231 (14)	0.0477 (5)	
H22	0.3672	0.3038	0.8281	0.057*	
C23	0.47292 (13)	0.11750 (14)	0.84941 (12)	0.0374 (4)	
C24	0.54190 (15)	0.14519 (15)	0.78829 (14)	0.0488 (5)	
H24	0.5195	0.1622	0.7342	0.059*	
C25	0.64357 (16)	0.14802 (18)	0.80620 (17)	0.0592 (6)	
H25	0.6889	0.1676	0.7647	0.071*	
C26	0.67731 (16)	0.12194 (17)	0.88523 (16)	0.0590 (6)	
H26	0.7458	0.1230	0.8971	0.071*	
C27	0.61055 (17)	0.09426 (16)	0.94707 (16)	0.0577 (6)	
H27	0.6339	0.0768	1.0007	0.069*	
C28	0.50785 (15)	0.09223 (15)	0.92967 (14)	0.0477 (5)	
H28	0.4627	0.0739	0.9718	0.057*	
C29	0.16603 (14)	−0.07883 (12)	0.79726 (11)	0.0348 (4)	
H29	0.2055	−0.1151	0.8385	0.042*	
C30	0.09214 (18)	−0.14639 (15)	0.75374 (14)	0.0520 (5)	
H30A	0.0463	−0.1710	0.7954	0.078*	
H30B	0.1287	−0.1972	0.7280	0.078*	
H30C	0.0552	−0.1133	0.7106	0.078*	
C31	0.23919 (13)	−0.04033 (13)	0.72887 (11)	0.0355 (4)	
H31A	0.2505	−0.0899	0.6874	0.043*	
H31B	0.2054	0.0105	0.6993	0.043*	
C32	0.34278 (14)	−0.00379 (13)	0.75768 (11)	0.0356 (4)	
H32	0.3806	0.0104	0.7056	0.043*	
C33	0.40092 (16)	−0.08103 (14)	0.80401 (15)	0.0497 (5)	
H33A	0.4652	−0.0572	0.8216	0.074*	
H33B	0.4104	−0.1334	0.7663	0.074*	
H33C	0.3637	−0.1009	0.8533	0.074*	

### Atomic displacement parameters (Å^2^)

**Table:**

	*U*^11^	*U*^22^	*U*^33^	*U*^12^	*U*^13^	*U*^23^
Cr1	0.02906 (13)	0.03611 (14)	0.02992 (12)	0.00083 (12)	−0.00075 (11)	−0.00429 (12)
P2	0.0271 (2)	0.0337 (2)	0.0320 (2)	0.00001 (19)	−0.00125 (17)	−0.00244 (18)
P3	0.0275 (2)	0.0372 (2)	0.0314 (2)	−0.00126 (18)	−0.00068 (18)	−0.00108 (19)
O1	0.0532 (10)	0.0809 (12)	0.0895 (13)	−0.0080 (9)	−0.0075 (9)	−0.0435 (11)
O2	0.0598 (9)	0.0915 (13)	0.0432 (8)	0.0009 (9)	0.0148 (7)	−0.0130 (8)
O3	0.0705 (11)	0.0641 (10)	0.0585 (9)	0.0125 (9)	0.0022 (8)	0.0236 (8)
O4	0.0799 (12)	0.0527 (10)	0.0763 (11)	0.0254 (9)	−0.0090 (10)	0.0008 (9)
C1	0.0381 (10)	0.0484 (11)	0.0480 (11)	0.0004 (9)	0.0006 (9)	−0.0132 (9)
C2	0.0410 (10)	0.0521 (12)	0.0370 (10)	0.0002 (9)	−0.0035 (8)	−0.0068 (9)
C3	0.0394 (10)	0.0519 (11)	0.0337 (9)	−0.0011 (9)	0.0015 (8)	0.0012 (8)
C4	0.0415 (11)	0.0472 (11)	0.0422 (10)	0.0042 (10)	−0.0019 (9)	−0.0086 (9)
C5	0.0335 (9)	0.0448 (10)	0.0362 (9)	0.0057 (8)	−0.0038 (8)	−0.0019 (8)
C6	0.0423 (10)	0.0425 (11)	0.0428 (10)	0.0046 (9)	−0.0039 (8)	0.0008 (9)
C7	0.0696 (15)	0.0531 (13)	0.0493 (12)	0.0118 (12)	−0.0021 (12)	0.0099 (11)
C8	0.0666 (15)	0.0781 (17)	0.0478 (12)	0.0153 (14)	−0.0230 (11)	−0.0005 (12)
C9	0.0440 (12)	0.0866 (18)	0.0569 (14)	−0.0002 (12)	−0.0165 (11)	−0.0009 (13)
C10	0.0398 (11)	0.0650 (14)	0.0491 (12)	−0.0049 (10)	−0.0078 (9)	0.0047 (11)
C11	0.0296 (8)	0.0541 (11)	0.0333 (9)	−0.0074 (8)	−0.0019 (8)	−0.0020 (9)
C12	0.0381 (10)	0.0736 (15)	0.0451 (11)	0.0030 (11)	0.0015 (9)	0.0009 (11)
C13	0.0354 (11)	0.124 (2)	0.0482 (12)	0.0020 (14)	0.0072 (10)	0.0005 (15)
C14	0.0499 (13)	0.125 (2)	0.0411 (11)	−0.0204 (16)	0.0055 (10)	0.0173 (15)
C15	0.0670 (16)	0.0786 (17)	0.0461 (12)	−0.0227 (14)	−0.0002 (12)	0.0155 (12)
C16	0.0479 (11)	0.0541 (12)	0.0406 (10)	−0.0079 (11)	0.0025 (9)	0.0027 (10)
C17	0.0290 (9)	0.0384 (10)	0.0394 (9)	−0.0028 (8)	0.0018 (8)	0.0020 (8)
C18	0.0485 (12)	0.0506 (11)	0.0465 (11)	−0.0083 (10)	−0.0059 (9)	0.0091 (9)
C19	0.0620 (14)	0.0724 (16)	0.0585 (13)	−0.0095 (14)	−0.0087 (12)	0.0269 (12)
C20	0.0587 (14)	0.0624 (15)	0.0818 (17)	0.0047 (12)	0.0057 (14)	0.0332 (14)
C21	0.0523 (13)	0.0380 (11)	0.0958 (19)	−0.0006 (10)	0.0202 (13)	0.0070 (12)
C22	0.0428 (11)	0.0426 (11)	0.0577 (12)	−0.0026 (9)	0.0070 (10)	−0.0020 (9)
C23	0.0286 (8)	0.0377 (9)	0.0458 (9)	0.0011 (8)	−0.0039 (7)	−0.0074 (9)
C24	0.0366 (10)	0.0571 (13)	0.0528 (12)	0.0000 (9)	−0.0012 (9)	−0.0013 (10)
C25	0.0312 (10)	0.0642 (15)	0.0822 (16)	−0.0029 (10)	0.0048 (11)	−0.0068 (13)
C26	0.0320 (10)	0.0591 (14)	0.0857 (16)	0.0036 (10)	−0.0140 (11)	−0.0225 (13)
C27	0.0479 (12)	0.0627 (14)	0.0624 (14)	0.0135 (11)	−0.0213 (11)	−0.0126 (11)
C28	0.0408 (10)	0.0550 (12)	0.0472 (11)	0.0045 (9)	−0.0063 (9)	−0.0056 (10)
C29	0.0337 (9)	0.0345 (9)	0.0362 (9)	−0.0024 (8)	0.0010 (8)	−0.0023 (7)
C30	0.0532 (12)	0.0475 (12)	0.0553 (12)	−0.0159 (10)	0.0072 (10)	−0.0128 (10)
C31	0.0350 (9)	0.0388 (10)	0.0327 (8)	−0.0024 (7)	0.0014 (7)	−0.0058 (7)
C32	0.0302 (8)	0.0393 (9)	0.0373 (9)	0.0003 (8)	0.0057 (7)	−0.0054 (8)
C33	0.0403 (11)	0.0421 (11)	0.0665 (14)	0.0091 (9)	−0.0008 (10)	−0.0081 (10)

### Geometric parameters (Å, °)

**Table:**

Cr1—C1	1.851 (2)	C16—H16	0.9300
Cr1—C2	1.8650 (19)	C17—C18	1.386 (3)
Cr1—C3	1.872 (2)	C17—C22	1.394 (3)
Cr1—C4	1.901 (2)	C18—C19	1.379 (3)
Cr1—P2	2.3736 (5)	C18—H18	0.9300
Cr1—P3	2.3847 (5)	C19—C20	1.357 (4)
P2—C17	1.8409 (19)	C19—H19	0.9300
P2—C23	1.8434 (17)	C20—C21	1.391 (4)
P2—C32	1.8680 (18)	C20—H20	0.9300
P3—C11	1.8352 (19)	C21—C22	1.379 (3)
P3—C5	1.8405 (18)	C21—H21	0.9300
P3—C29	1.8637 (18)	C22—H22	0.9300
O1—C1	1.152 (2)	C23—C24	1.384 (3)
O2—C2	1.144 (2)	C23—C28	1.388 (3)
O3—C3	1.146 (2)	C24—C25	1.382 (3)
O4—C4	1.138 (2)	C24—H24	0.9300
C5—C6	1.380 (3)	C25—C26	1.368 (3)
C5—C10	1.397 (3)	C25—H25	0.9300
C6—C7	1.384 (3)	C26—C27	1.372 (3)
C6—H6	0.9300	C26—H26	0.9300
C7—C8	1.382 (3)	C27—C28	1.393 (3)
C7—H7	0.9300	C27—H27	0.9300
C8—C9	1.367 (3)	C28—H28	0.9300
C8—H8	0.9300	C29—C30	1.535 (3)
C9—C10	1.391 (3)	C29—C31	1.548 (2)
C9—H9	0.9300	C29—H29	0.9800
C10—H10	0.9300	C30—H30A	0.9600
C11—C16	1.389 (3)	C30—H30B	0.9600
C11—C12	1.395 (3)	C30—H30C	0.9600
C12—C13	1.389 (3)	C31—C32	1.540 (2)
C12—H12	0.9300	C31—H31A	0.9700
C13—C14	1.376 (4)	C31—H31B	0.9700
C13—H13	0.9300	C32—C33	1.528 (3)
C14—C15	1.371 (4)	C32—H32	0.9800
C14—H14	0.9300	C33—H33A	0.9600
C15—C16	1.392 (3)	C33—H33B	0.9600
C15—H15	0.9300	C33—H33C	0.9600
			
C1—Cr1—C2	87.81 (8)	C18—C17—C22	118.39 (18)
C1—Cr1—C3	91.82 (9)	C18—C17—P2	124.08 (15)
C2—Cr1—C3	88.86 (9)	C22—C17—P2	117.33 (15)
C1—Cr1—C4	89.84 (9)	C19—C18—C17	120.5 (2)
C2—Cr1—C4	87.52 (9)	C19—C18—H18	119.7
C3—Cr1—C4	175.96 (9)	C17—C18—H18	119.7
C1—Cr1—P2	88.80 (6)	C20—C19—C18	121.0 (2)
C2—Cr1—P2	176.35 (6)	C20—C19—H19	119.5
C3—Cr1—P2	89.89 (6)	C18—C19—H19	119.5
C4—Cr1—P2	93.83 (6)	C19—C20—C21	119.7 (2)
C1—Cr1—P3	178.55 (7)	C19—C20—H20	120.2
C2—Cr1—P3	91.96 (6)	C21—C20—H20	120.2
C3—Cr1—P3	86.74 (6)	C22—C21—C20	119.8 (2)
C4—Cr1—P3	91.58 (6)	C22—C21—H21	120.1
P2—Cr1—P3	91.389 (18)	C20—C21—H21	120.1
C17—P2—C23	99.78 (9)	C21—C22—C17	120.6 (2)
C17—P2—C32	105.87 (8)	C21—C22—H22	119.7
C23—P2—C32	99.66 (8)	C17—C22—H22	119.7
C17—P2—Cr1	112.20 (6)	C24—C23—C28	118.61 (17)
C23—P2—Cr1	118.79 (6)	C24—C23—P2	119.95 (14)
C32—P2—Cr1	118.14 (6)	C28—C23—P2	121.31 (15)
C11—P3—C5	102.74 (9)	C25—C24—C23	121.1 (2)
C11—P3—C29	105.52 (9)	C25—C24—H24	119.4
C5—P3—C29	99.48 (9)	C23—C24—H24	119.4
C11—P3—Cr1	109.25 (6)	C26—C25—C24	119.8 (2)
C5—P3—Cr1	123.28 (7)	C26—C25—H25	120.1
C29—P3—Cr1	114.67 (6)	C24—C25—H25	120.1
O1—C1—Cr1	178.18 (18)	C25—C26—C27	120.3 (2)
O2—C2—Cr1	176.29 (17)	C25—C26—H26	119.8
O3—C3—Cr1	177.93 (18)	C27—C26—H26	119.8
O4—C4—Cr1	176.72 (18)	C26—C27—C28	120.1 (2)
C6—C5—C10	118.50 (18)	C26—C27—H27	119.9
C6—C5—P3	118.16 (14)	C28—C27—H27	119.9
C10—C5—P3	122.65 (16)	C23—C28—C27	120.0 (2)
C5—C6—C7	121.2 (2)	C23—C28—H28	120.0
C5—C6—H6	119.4	C27—C28—H28	120.0
C7—C6—H6	119.4	C30—C29—C31	108.47 (15)
C8—C7—C6	119.8 (2)	C30—C29—P3	113.99 (14)
C8—C7—H7	120.1	C31—C29—P3	110.76 (13)
C6—C7—H7	120.1	C30—C29—H29	107.8
C9—C8—C7	119.9 (2)	C31—C29—H29	107.8
C9—C8—H8	120.1	P3—C29—H29	107.8
C7—C8—H8	120.1	C29—C30—H30A	109.5
C8—C9—C10	120.7 (2)	C29—C30—H30B	109.5
C8—C9—H9	119.7	H30A—C30—H30B	109.5
C10—C9—H9	119.7	C29—C30—H30C	109.5
C9—C10—C5	119.9 (2)	H30A—C30—H30C	109.5
C9—C10—H10	120.0	H30B—C30—H30C	109.5
C5—C10—H10	120.0	C32—C31—C29	118.62 (15)
C16—C11—C12	118.76 (19)	C32—C31—H31A	107.7
C16—C11—P3	122.94 (15)	C29—C31—H31A	107.7
C12—C11—P3	117.91 (16)	C32—C31—H31B	107.7
C13—C12—C11	120.6 (2)	C29—C31—H31B	107.7
C13—C12—H12	119.7	H31A—C31—H31B	107.1
C11—C12—H12	119.7	C33—C32—C31	110.44 (16)
C14—C13—C12	119.6 (2)	C33—C32—P2	111.66 (13)
C14—C13—H13	120.2	C31—C32—P2	114.61 (12)
C12—C13—H13	120.2	C33—C32—H32	106.5
C15—C14—C13	120.6 (2)	C31—C32—H32	106.5
C15—C14—H14	119.7	P2—C32—H32	106.5
C13—C14—H14	119.7	C32—C33—H33A	109.5
C14—C15—C16	120.2 (3)	C32—C33—H33B	109.5
C14—C15—H15	119.9	H33A—C33—H33B	109.5
C16—C15—H15	119.9	C32—C33—H33C	109.5
C11—C16—C15	120.2 (2)	H33A—C33—H33C	109.5
C11—C16—H16	119.9	H33B—C33—H33C	109.5
C15—C16—H16	119.9		
			
C1—Cr1—P2—C17	−87.62 (9)	C13—C14—C15—C16	−0.2 (4)
C3—Cr1—P2—C17	−179.44 (9)	C12—C11—C16—C15	1.3 (3)
C4—Cr1—P2—C17	2.15 (9)	P3—C11—C16—C15	173.94 (17)
P3—Cr1—P2—C17	93.82 (7)	C14—C15—C16—C11	−0.6 (3)
C1—Cr1—P2—C23	28.07 (10)	C23—P2—C17—C18	121.48 (17)
C3—Cr1—P2—C23	−63.75 (10)	C32—P2—C17—C18	18.42 (19)
C4—Cr1—P2—C23	117.84 (10)	Cr1—P2—C17—C18	−111.79 (16)
P3—Cr1—P2—C23	−150.49 (8)	C23—P2—C17—C22	−63.80 (17)
C1—Cr1—P2—C32	148.80 (10)	C32—P2—C17—C22	−166.86 (15)
C3—Cr1—P2—C32	56.98 (9)	Cr1—P2—C17—C22	62.93 (16)
C4—Cr1—P2—C32	−121.44 (9)	C22—C17—C18—C19	−2.1 (3)
P3—Cr1—P2—C32	−29.76 (7)	P2—C17—C18—C19	172.58 (18)
C2—Cr1—P3—C11	−23.62 (10)	C17—C18—C19—C20	1.5 (4)
C3—Cr1—P3—C11	65.13 (9)	C18—C19—C20—C21	0.2 (4)
C4—Cr1—P3—C11	−111.19 (9)	C19—C20—C21—C22	−1.3 (4)
P2—Cr1—P3—C11	154.94 (7)	C20—C21—C22—C17	0.7 (3)
C2—Cr1—P3—C5	96.96 (10)	C18—C17—C22—C21	1.0 (3)
C3—Cr1—P3—C5	−174.29 (9)	P2—C17—C22—C21	−174.05 (17)
C4—Cr1—P3—C5	9.39 (10)	C17—P2—C23—C24	−33.81 (18)
P2—Cr1—P3—C5	−84.48 (8)	C32—P2—C23—C24	74.30 (18)
C2—Cr1—P3—C29	−141.81 (9)	Cr1—P2—C23—C24	−155.96 (14)
C3—Cr1—P3—C29	−53.06 (9)	C17—P2—C23—C28	150.40 (17)
C4—Cr1—P3—C29	130.62 (9)	C32—P2—C23—C28	−101.49 (17)
P2—Cr1—P3—C29	36.75 (7)	Cr1—P2—C23—C28	28.25 (19)
C11—P3—C5—C6	164.12 (15)	C28—C23—C24—C25	−0.1 (3)
C29—P3—C5—C6	−87.46 (16)	P2—C23—C24—C25	−176.02 (18)
Cr1—P3—C5—C6	40.56 (18)	C23—C24—C25—C26	0.9 (4)
C11—P3—C5—C10	−25.5 (2)	C24—C25—C26—C27	−0.9 (4)
C29—P3—C5—C10	82.87 (18)	C25—C26—C27—C28	0.2 (4)
Cr1—P3—C5—C10	−149.11 (15)	C24—C23—C28—C27	−0.6 (3)
C10—C5—C6—C7	−3.0 (3)	P2—C23—C28—C27	175.27 (16)
P3—C5—C6—C7	167.71 (16)	C26—C27—C28—C23	0.5 (3)
C5—C6—C7—C8	2.5 (3)	C11—P3—C29—C30	56.18 (16)
C6—C7—C8—C9	−0.6 (4)	C5—P3—C29—C30	−49.99 (16)
C7—C8—C9—C10	−0.8 (4)	Cr1—P3—C29—C30	176.46 (12)
C8—C9—C10—C5	0.2 (4)	C11—P3—C29—C31	178.84 (12)
C6—C5—C10—C9	1.7 (3)	C5—P3—C29—C31	72.67 (14)
P3—C5—C10—C9	−168.63 (18)	Cr1—P3—C29—C31	−60.89 (13)
C5—P3—C11—C16	134.04 (16)	C30—C29—C31—C32	−156.37 (17)
C29—P3—C11—C16	30.26 (18)	P3—C29—C31—C32	77.82 (19)
Cr1—P3—C11—C16	−93.51 (16)	C29—C31—C32—C33	58.3 (2)
C5—P3—C11—C12	−53.21 (17)	C29—C31—C32—P2	−68.9 (2)
C29—P3—C11—C12	−156.99 (15)	C17—P2—C32—C33	151.59 (14)
Cr1—P3—C11—C12	79.24 (15)	C23—P2—C32—C33	48.44 (15)
C16—C11—C12—C13	−1.1 (3)	Cr1—P2—C32—C33	−81.72 (14)
P3—C11—C12—C13	−174.20 (17)	C17—P2—C32—C31	−81.88 (14)
C11—C12—C13—C14	0.3 (4)	C23—P2—C32—C31	174.97 (14)
C12—C13—C14—C15	0.4 (4)	Cr1—P2—C32—C31	44.80 (15)
